# Medico-Chirurgical Transactions, Vol. XVII

**Published:** 1833-01-01

**Authors:** 


### Medico-Chirurgical Transactions, published by the Medical and Chirurgical Society of London. Volume the Seventh. 8vo. pp. 513. Two Lithographic Plates. London, 1832.

The present volume of the Society's Transactions, though not so valuable as some of its predecessors, contains good papers, and offers on the whole a fair specimen of the present state of knowledge among the best informed members of our profession. When we state that in the array of contributors, are Mr. Brodie, Mr. Lawrence, Mr. Travers, Mr. Samuel Cooper, Mr. Langstaff, Mr. Cæsar Hawkins, Dr. Hodgkin, and Dr. Lee, we offer a tolerable guarantee for the quality of much of the matter. We think it highly desirable that this Society, or at all events, one of a similar kind, should be supported. It is a point d'appui for our best men, and the publication of its Transactions is calculated to benefit science, and maintain our professional character abroad. It would not be difficult to shew that the publication of such transactions is more calculated to attain the ends to which we have adverted, than the practice of sending communications to periodical journals. They appear so frequently, and contain matter of such various quality, that after some years it would become a terrific affair to refer to their many volumes and multitudinous pages for any particular paper. The Medico-Chirurgical Society is to us a sort of Amphictyonic Council, where the deputies from the various departments of our profession meet. It certainly is not to be compared to the Institute or Académie des Sciences, of France, but it serves as a substitute for such establishments, and we do believe that its decay would be a serious evil.

We shall proceed without further preface to notice as many of the papers as our limited space will permit. One of the most valuable, though, as usual, the least pretending, is from Mr. Brodie. The subject is abscess in the interior of the tibia. The communication is too brief and too concise to admit of much further abbreviation on our part, and we feel no regret at this, the paper displaying Mr. Brodie's characteristic method, precision and clearness.

"I am not aware," says Mr. Brodie, "that any cases exactly similar to those which I am about to relate have been recorded by authors: and as they appear to me to throw some light on the history and treatment of a rare but very serious disease, I am led to believe that they are not unworthy of being communicated to the Medical and Chirurgical Society." 239.

*Case* 1. Mr. P. æt. 24, consulted Mr. Brodie in October, 1824, under the following circumstances.

"There was a considerable enlargement of the lower extremity of the right tibia, extending to the distance of two or three inches from the ankle-joint. The integuments at this part were tense, and they adhered closely to the surface of the bone.

The patient complained of a constant pain referred to the enlarged bone and neighbouring parts. The pain was always sufficiently distressing; but he was also liable to more severe paroxysms in which his sufferings were described as most excruciating. These paroxysms recurred at irregular intervals, confining him to his room for many successive days, and being attended with a considerable degree of constitutional disturbance. Mr. P. described the disease as having existed more than twelve years, and as having rendered his life miserable during the whole of that period.

In the course of this time he had been under the care of various surgeons, and various modes of treatment had been resorted to without any permanent advantage. The remedies which I prescribed for him were equally inefficacious. Finding himself without any prospect of being relieved by other means, he made up his mind to lose the limb by amputation; and Mr. Travers having seen him with me in consultation, and having concurred in the opinion, that this was the best course which could be pursued, the operation was performed accordingly." 240.

The patient died on the fifth day after the operation, with those nervous symptoms, resembling delirium tremens, which we see occur occasionally after injuries or operations.

"On examining the amputated limb, it was found that a quantity of new bone had been deposited on the surface of the lower extremity of the tibia. This deposition of new bone was manifestly the result of inflammation of the periosteum at some former period. It was not less than one-third of an inch in thickness, and when the tibia was divided longitudinally with a saw, the line at which the new and old bone were united with each other, was distinctly to be seen.

The whole of the lower extremity of the tibia was harder and more compact than under ordinary circumstances, in consequence, as it appeared, of some deposit of bone in the cancellous structure, and in its centre, about one-third of an inch above the ankle, there was a cavity of the size of an ordinary walnut, filled with a dark-coloured pus. The bone immediately surrounding this cavity, was distinguished from that in the neighbourhood by its being of a whiter colour, and of a still harder texture, and the inner surface of the cavity presented an appearance of high vascularity. The ankle-joint was free from disease." 242.

This case made a strong impression on Mr. Brodie. It seemed evident to him that, had its nature been understood, and the pus locked up in the bone been let out by means of the trephine, the patient might have preserved both his limb and his life. In the course of two years another case presented itself.

*Case* 2. Mr. B. æt. 23, consulted Mr. Brodie in February, 1826.

"There was a considerable enlargement of the right tibia, beginning immediately below the knee, and extending downwards so as to occupy about one-third of the length of the bone.

Mr. B. complained of excessive pain, which disturbed his rest at night, and some parts of the swelling were tender to the touch. The knee itself was not swollen, and its motions were perfect.

He said that the disease had begun more than ten years ago, with a slight enlargement and pain in the upper extremity of the tibia; and that these symptoms had gradually increased up to the time of my being consulted. Various remedies had been employed, from which, however, he had derived little or no advantage." 243.

Mr. Brodie looked upon the case as one of chronic periostitis, and treated it as such by an incision through the periosteum, and the subsequent exhibition of sarsaparilla internally. The periosteum was found much thickened, and the new bone soft and vascular. The pain was immediately relieved, the wound gradually healed, and the patient was supposed to be cured. But the enlargement of the upper end of the tibia never entirely subsided, and in August, 1827, pain returned in it. The pain gradually increased, and in January, 1828, Mr. Brodie was again consulted. The pain was constant, yet more severe at one time than another, and often preventing sleep for several successive nights—the enlargement of the tibia as great as ever—the skin tense and unnaturally adherent to the bone—the patient unable to follow his usual avocations. The resemblance to the former case struck Mr. Brodie; he proposed trephining the tibia, the patient consented, and in March, 1828, the operation was performed.

"My attention was directed to a spot about two inches below the knee, to which the pain was particularly referred. This part of the tibia was exposed by a crucial incision of the integuments. The periosteum now was not in the same state as at the time of the former operation. It was scarcely thicker than natural, and the bone beneath was hard and compact. A trephine of a middle size was applied, and a circle of bone was removed extending into the cancellous structure, but no abscess was discovered. I then, by means of a chisel, removed several other small portions of bone at the bottom of the cavity made by the trephine. As I was proceeding in this part of the operation the patient suddenly experienced a sensation, which he afterwards described as being similar to that which is produced by touching the cavity of a carious tooth, but much more severe, and immediately some dark-coloured pus was seen to issue slowly from the part to which the chisel had been last applied. This was absorbed by a sponge, so that the quantity of pus which escaped was not accurately measured, but it appeared to amount in all to about two drams. From this instant the peculiar pain belonging to the disease entirely ceased, and it has never returned. The patient experienced a good deal of pain, the consequence of the operation, for the first twenty-four hours, after which there was little or no suffering. The wound was dressed lightly to the bottom with lint. Nearly six months elapsed before it was completely cicatrized: but in about three months from the day of the operation, Mr. B. was enabled to walk about and attend to his usual occupations. He has continued well to the present time (January 7, 1832;) and the tibia is now reduced in size so as to be scarcely larger than that of the other leg. No exfoliation of bone has ever taken place." 246.

*Case* 3. In January, 1830, Mr. S. æt. 24, consulted Mr. Brodie.

"The lower extremity of the left tibia was considerably enlarged; the skin covering it was tense, and adhered closely to the parts below. The patient complained of a constant aching pain, which he referred to the enlarged bone. Once in two or three weeks there was an attack of pain more severe than usual, during which his sufferings were excruciating, lasting several hours, and sometimes one or two days, and rendering him altogether incapable of following his usual occupations. The pain was described as shooting and throbbing, worse during the night, and attended with such exquisite tenderness of the parts in the neighbourhood of the ankle that the slightest touch was intolerable.

Mr. S. said that, to the best of his recollection, the disease had begun eighteen years ago, in the following manner. On going to bed one evening he suddenly experienced a most acute pain in the inner ankle. On the following morning he was unable to put his foot to the ground, on account of the agony which every attempt to do so occasioned. Leeches were applied several times, and afterwards blisters, but the pain increased notwithstanding. After some weeks an abscess presented itself and broke. This was followed by some mitigation of the symptoms. Soon afterwards another abscess formed and broke in the neighbourhood of the first. The two abscesses remained open for a considerable time, and then healed rapidly. Mr. S. now began to regain the use of the limb, and by degrees was able to walk as usual.

During the following summer he had a recurrence of pain in the inner ankle, without any further formation of abscess. For eight or ten years afterwards there were occasional attacks of pain, lasting one or two days at a time; the intervals between them being of various duration, and in one instance, not less than nine months. After this the attacks recurred more frequently, and during the whole of the last two years the symptoms were nearly as severe as at the time of my being consulted.

On examining the limb I was struck with the resemblance which it bore to that of the limb in each of the two preceding cases. There was also a remarkable resemblance in the symptoms as described by the patient, and I could not but suspect that they depended on a similar cause. I requested that Mr. Travers, who had attended one of the former cases with me, should be consulted: and he agreed with me in the opinion that probably an abscess existed in the centre of the tibia, and that it would be advisable to perforate the bone with a trephine, with the view of enabling the contents of the abscess to escape.

Accordingly I performed the operation, with the assistance of Mr. Travers, on the 31st of January. A crucial incision was made through the skin, the angles of which were raised so as to expose a part of the bone above the inner ankle, to which the pain was especially referred. A small trephine was then applied, and a circular portion of bone was removed extending into the cancellous structure. Other portions of bone were removed with a narrow chisel. At last about a dram of pus suddenly escaped and rose into the opening made by the trephine and chisel. On further examination a cavity was discovered from which the pus had flowed, capable of admitting the extremity of the finger. The inner surface of this cavity was exquisitely tender; the patient experiencing the most excruciating pain on the gentlest introduction of the probe into it." 248.

Soon after the operation some inflammation occurred, an abscess formed between the periosteum and the bone, and subsequently other abscesses presented themselves in the neighbourhood. But all healed favourably—the cavity made by the trephine became filled up—the wound gradually cicatrized—the pain formerly felt never re-appeared—and the patient was soon able to resume his former pursuits. He continues well.

It is singular that an affection of this nature, of which samples must have occurred in the practice of many surgeons, should now, for the first time, have been clearly described. In point of fact, it has long been known that an abscess may occur in the medullary cavity, or the cancellated ends of long bones. In Mr. Bloomfield's "Chirurgical Cases," we find the following remarks on the spina ventosa, or as he chooses, with the quaint pedantry of his time to call it, "the abscessus in the medulla."

"It is universally allowed, that this disease takes its rise from matter being formed either in the *diploe*, or in the marrow; whenever obstruction is begun in the vessels expanded on, or terminating in, the medullary *cysts*, the consequence will be inflammation, and, if not early removed, will form matter; for this reason, I generally called this case *abscessus in medulla*. Whenever then a patient complains of a dull, heavy pain, deeply situated in the bone, possibly consequent to a violent blow received on the part some time before, and though at the time the patient complains of this uneasiness, within the bone, the integuments shall appear perfectly sound, and the bone itself not in the least injured, we have great reason to suspect an *abscessus* in the *medulla*. Children of a bad habit of body, though they have not suffered any external injury, will often become lame, and complain of the limb being remarkably heavy, and though not attended with acute pain, yet, the dull, throbbing uneasiness, is constant. If rigors happen during the time the patient labours under this indisposition, it generally implies that matter will be formed within the substance of the bone. On the age of the patient, and the solidity of the bone, will in a great measure depend the next alarming symptoms, to those who are not acquainted thoroughly with the case, and the constitution of the patient, as mothers and nurses are often inattentive to children on their first complaining of pain, and heaviness in a limb, if after rubbing it with their hand a few minutes, the child, amused by some new toy or play-fellow, think himself easier, the good women, as they cannot see any thing wrong, determine it *a growing pain*, as they call it; but, soon after, the extremities of the bone formerly complained of, begin to swell, or, possibly throughout its whole extent, it becomes inlarged; a surgeon is then sent for, who, if, a man of experience, will know this to be an *abscessus in medulla*, or true *spina ventosa*, as it is called: if neither of these symptoms should be consequent to the first complaint, the great insensibility of the bone in some subjects, will prevent that acuteness of pain usual in other parts, where matter is formed, though the acrid matter is eroding the bone during the whole time it is contained within it. This matter at length having made its way through, arrives at the *periosteum*, where it creates most violent pain, as well from its sharpness, as from its increased quantity, occasioning an extension of the membrane, which I declare *inelastic*. The integuments then become swelled and inflamed, and have a sort of emphysematous feel. On being examined, by pressure, the tumor will sometimes be lessened, from part of the matter retiring into the bone: from this appearance to the touch, most likely the name of *ventosa* was added to the term *spina*. When we are assured of matter being under the *periosteum*, we cannot be too early in letting it out, as it will save a considerable deal of pain to the patient, though probably it may not be of any considerable advantage in respect to the carious bone; for, as I have more than once before observed, where the fluids in general are vitiated, no chance of cure can be expected from topical remedies, but where the constitution is mended, nature will sometimes astonish us in her part; as the carious bone will be thrown off from the *epiphyses*, or, the *teredines* will be filled up by the ossific matter, that flows from the parts of the bone, where some of the *spinæ* have come away.

If proper medicines are given, the children well supported, and the parts kept clean and dry, *patience* and *perseverance* will frequently give great credit to the surgeon. And from my dislike to amputate, where the disorder is not *merely local*, I have had my proportion of honor *in perfecting a surprizing cure*, without any farther pretensions to superior skill than what I have just related. In case it should have been thought advisable to apply the head of a *trephine* at the upper and lower extremities of the *tibia*, to give free discharge to the matter, the washing *it* away, as well as the small crumblings of the carious bone, by means of detersive and drying injections, I have known to be greatly contributory to the curing this kind of *caries*, after the habit of body in general had been mended."

Though Mr. Bloomfield had nearly stumbled on the truth, it is evident that he has not hit it. He has mixed up in one description several diseases of bone, which modern investigation has distinguished. The surgeon who went into practice with Mr. Bloomfield's notions of an "abscessus in the medulla," and he who had perused Mr. Brodie's straight-forward account of "some cases of chronic abscess in the tibia," would probably be practitioners of a very opposite sort. By the way, this is no bad commentary on other remarks of Mr. Bloomfield, who, if we may venture to form an opinion from his writings, was possessed of no unphilosophical mind. In speaking of the benefits accruing from the establishment of hospitals, he uses the following expressions.

"Though under these advantages almost every operation in surgery has been so much improved, and so plainly taught, that, as it may seem to some men, scarce any thing further is to be expected, yet the experience of every day may convince us that there is still a large field to traverse, which has not yet been trodden."

This was in 1773. Could Mr. Bloomfield revisit his own hospital, (he was surgeon to St. George's) he would see in the noble construction of the new building, the symbol of the improvements in surgery which himself had so sagaciously anticipated.

In Mr. Hey's remarks upon caries of the tibia, and the employment of the trephine for its removal, two cases are related in which there was abscess in the tibia. In both, however, the matter had made its way through the anterior lamella of the tibia by the process of ulceration, prior to the performance of the operation of trephining. It is curious that Mr. Hey, a practical man, reasoned very justly on the progress of the cases, and adopted a very sensible and successful operation for the cure of the disease, yet did not appear to suspect that in some instances it might be necessary or proper to perforate the tibia prior to the occurrence of any ulcerated opening. In Mr. Hey's cases the ulcerative action prevailed, in the cases related by Mr. Brodie the inflammatory process was limited to the deposition of new ossific matter, and consolidation of the cancellous tissue round the abscess. We have not at present by us the little work of Mr. Benjamin Bell on the diseases of bones. If our memory does not deceive us, that gentleman gives a plate of an abscess in the lower end of the tibia, walled in by new bone, as in Mr. Brodie's cases. We think too it is hinted that a perforation of the bone by the trephine would have been desirable, but nothing further is said. In short, we know of no satisfactory account of these chronic abscesses of the tibia with the exception of Mr. Brodie's, and we recommend a careful consideration of his cases to all practical surgeons.

### II. SOME EXPERIMENTS ON THE USE OF STYPTICS IN HÆMORRHAGE FROM ARTERIES. By *Cæsar Hawkins*, Esq.

Some little time ago MM. Talrich and Halmagrand arrived in this country from France with a styptic, the composition of which they kept secret, whilst they freely offered to display its effects. Previously to their arrival, their experiments and their conclusions had been noticed in the Parisian journals, and they brought with them a printed exposé of their views, the substance of a paper read by them, we believe, to the Academy. We took no notice of the styptic or its advocates at the time, because we could not consent to countenance the humbug, native or foreign, of a secret remedy. We witnessed some of the experiments performed by the gentlemen to whom we have alluded, and we can only say that on the whole they were more calculated to encourage suspicion of the remedy and its proposers, than to inspire much confidence in either. We say this with no wish to hurt the feelings or prejudice the interests of Messrs. Talrich and Halmagrand. We know that they are very ingenious and can readily believe them highly honourable, but they went the wrong way to work to establish their views, or remove all suspicion as to their motives.

Foreign surgeons are still much behind us in their treatment of wounds, and in their surgical management of arteries. We have only to cast our eyes over a few French journals, to be satisfied of this. MM. Talrich and Halmagrand were, therefore, unfortunate in coming to a country where sounder notions on the subject of hæmorrhage prevailed, and where some one would infallibly arise to point out any sophistry in which they might indulge, or errors into which they might fall. Such an one did arise, and Mr. Hawkins was he. When we say that the profession should be obliged to Mr. Hawkins for placing the matter in its proper light, for vindicating and confirming the views, and for directing attention to the experiments of Dr. Jones, we are sure we only echo a general sentiment. Mr. Hawkins observes that some persons were deceived who ought to have known better. Certainly some of our contemporaries were in too great a hurry to celebrate the renown of MM. the French experimentalists. As it is better to diffuse sound notions than unsound, we shall dedicate some of that space to the consideration of Mr. Hawkins' paper, which might have been less profitably devoted to the "false gods" of the Frenchmen.

"In most of Dr. Jones's experiments the free passage of the blood from the wounded artery was nearly, or completely, prevented by a continued suture in the skin, so that the blood necessarily forced its way into the cellular texture around, and there became coagulated; a method which he adopted from having found in some earlier experiments, that without this precaution the sudden loss of blood was generally fatal before the necessary clots could be formed. The mode in which, on the other hand, M. Halmagrand conducts his experiments is, to place three small tampons, or compresses of cotton dipped in his 'Liquide hæmostatique,' upon the wound of the artery, and retain them there for ten minutes with the fingers, after which a *single ligature* only through the skin is employed, which does not therefore close the wound, but merely prevents the compresses from being thrown out of the wound by muscular effort; a precaution which he has found necessary during the exertion of the animal to rise, and the omission of which occasioned fatal hæmorrhage in one of his experiments, at which I was present. This method I exactly imitated in the following experiments."

*Experiment* 1. "I exposed the carotid artery of a sheep, and made in it a longitudinal wound about half an inch in length, and placed upon the orifice compresses of tow, of the same size as those which I had seen employed by M. Halmagrand, dipped in a decoction of *Aleppo galls,* (one ounce having been boiled for ten minutes in three-fourths of a pint of water,) and tied a single ligature in the skin before I withdrew my fingers from the wound. The animal was restless during the experiment, in consequence of which, perhaps, a small quantity of blood (about half an ounce) escaped when it rose from the ground, but none was lost subsequently. The compresses were taken away on the second day, and the sheep was killed on the tenth day by another experiment.

The wound in the artery was perfectly closed by lymph, without the formation of any coagulum in the vessel, which consequently remained pervious, the cicatrix itself being hardly perceptible. Some coagulum had formed externally along the course of the artery, in the same manner as it had taken place in one of the sheep in which M. Halmagrand had made a longitudinal wound and afterwards applied his styptic, killing it, however, on the fifth day. (See Preparation, No. 1."* 125.

* There are no plates appended to Mr. Hawkins' paper. The preparations

*Experiment* 2. M. Halmagrand laid bare the carotid artery of another sheep in his usual manner. Mr. Hawkins then divided the artery transversely to exactly half its circumference, the most fatal description of wound. Owing to a mistake on the part of M. Halmagrand, the animal lost a pint and a half of blood, and fainted. When it revived, the artery again began to bleed freely. Mr. Hawkins then placed on the artery the usual compresses, dipped in Ruspini's styptic (a good quack medicine or application), and held them for ten minutes, using a single ligature in the skin. No hæmorrhage followed, the compresses were removed at the same time as the first sheep (that we were told was on the *second* day), and the animal was killed by another experiment on the *eighth* day.

"There was some extravasation in the course of the vessel; the external wound, which was suppurating, communicated directly with the artery, which was completely blocked up by a firm coagulum, united by lymph to the interior of the vessel for about half an inch above and below the opening, connected on the side next to the heart with a loose coagulum two inches long. (See preparation No. 2 )" 126.

*Experiment* 3. Mr. Hawkins exposed the carotid artery of a sheep, cut out a circular portion of the vessel, and placed over the opening the usual compresses, dipped in plain water only. The animal was restless, and blood escaped several times during its struggles. After some time the compresses were removed, when the blood issued in as large a stream as at first. Fresh compresses, dipped in water, were applied and retained for ten minutes, the animal still struggling. When the pressure was relaxed, the vessel still bled (in a similar experiment M. Halmagrand was unsuccessful); Mr. Hawkins altered his plan, and united the skin by a coarse continued suture. As soon as the fingers were removed, the integuments were distended with blood, and some escaped between the loose stitches. The animal got up, there was no further hæmorrhage, and it was killed on the sixth day, the wound being then open, and suppurating. The interior of the vessel was filled with coagulum, considerably above and below the wound, which was blocked up by a portion projecting into the aperture—(*See* Preparation, No. 3.)

*Experiment* 4. "I exposed the right carotid artery of the sheep, in which the left carotid had been opened in Experiment II., and removed a portion with scissors, and then placed on the wound three compresses of the usual size dipped in *plain water*, and made pressure with the fingers for ten minutes, and then put a single ligature through the skin. The animal lay quiet during the experiment, and not a drop of blood was lost till the next day, when considerable hæmorrhage occurred, and this continued at intervals till the third day, when it died from the bleeding.

The wound of the soft parts was so small and direct that no extravasation had taken place into the sheath of the vessel, and no external coagulum had formed, so that the wound of the vessel remained open. Within it a small recent coagulum only had formed, which nearly closed the end of the vessel nearest to the head, but left the lower orifice quite open. (See preparation No. 4.)" 128.

There was reason to believe that the animal disturbed the compresses by endeavouring to drink from a high pail.

---

referred to are some very neatly put up in spirit, and preserved in the museum of St. George's Hospital. They fully bear out the text.

*Experiment* 5. Mr. Hawkins exposed the left carotid, in the sheep which had been the subject of the first experiment, and placed a hook under the vessel. He then coarsely sewed up the wound in the skin by the continued suture, leaving room for the vessel to be drawn to the edge, and removed with the scissors a portion of the vessel, equal to nearly half its circumference. The hook was then withdrawn, the vessel allowed to recede, the ligature tightened, so as nearly to prevent the exit of the blood, which came through the wound copiously for a few seconds, and then ceased. No further hæmorrhage occurred till the third day, when some dropped from the wound, and continued to do so at intervals till the fourth day, when the animal died. The wound was much distended with coagulum, and suppuration was commencing—there was no plug around the opening, nor any sufficient clot in the interior of the artery.

"I should observe, that in all probability the last two experiments were performed under unfavourable circumstances, as the opposite carotid arteries had already been operated on, and in Experiment 4, the left carotid having been entirely obstructed by Experiment 2, only the vertebral arteries remained to convey blood to the head, so that probably the impulse through the right carotid became more powerful than it otherwise would have been; but, on the other hand, this very circumstance, which may have contributed to the unsuccessful result of Experiments 4 and 5, renders the success of Experiments 1 and 2 still more striking." 129.

The following are the conclusions drawn by Mr. Hawkins from the preceding experiments. They appear to be fully warranted by them, and fairly deducible from them. Yet there seems to be some obscurity about one inference drawn by Mr. Hawkins. He says that one experiment alone, viz. Exper. 4, was altogether unsuccessful, "the animal having, in this case, only died of the operation." In another place, he observes that "Experiments 3 and 5 shew, that when a portion of an artery has been altogether removed, the hæmorrhage may be restrained, and the vessel permanently closed, by allowing the blood to coagulate in the soft parts, as in a diffused aneurism." It may prove great stolidity on our part, but we must confess that we do not exactly perceive how Experiment 5 bears out this assertion. In that experiment, hæmorrhage occurred on the third day, and continued at intervals till the fourth, when the animal died. It does appear to us, that in this case the operation was the cause of death. The difference between this and the fourth experiment is merely a matter of time; for in Exper. 4, the hæmorrhage occurred on the day after the operation, and continued at intervals till the third, when the animal died. If one died from the operation, so, as it seems to us, did the other. How, again, can Experiment 5 be said to shew, that the vessel may be *permanently* closed after the removal of a portion of it, when, as we have shewn, the animal died of hæmorrhage, and when, as Mr. Hawkins informs us, "the opening of the artery was undefended by any plug, neither was there any coagulum in the interior of the artery, sufficiently large to prevent hæmorrhage from either side?" These inferences from Experiment 5 do seem to be irreconcileable with a correct induction, and we point out the apparent inconsistencies, in order that Mr. Hawkins may clear them up, as no doubt he can. With this remark we subjoin the conclusions of Mr. Hawkins, as deserving of attention from those who would be possessed of accurate knowledge of the natural means of arresting hæmorrhage.

"It appears, then, that in all these varied experiments, the flow of blood was restrained for a time, the shortest period at which secondary hæmorrhage occurred having been twenty or twenty-four hours, and that in three of them the wound in the vessel was adequately closed to have prevented any further hæmorrhage. It appears, also, that out of *four* experiments performed in the same manner as M. Halmagrand's, but without the use of his styptic, *two*, viz. Experiments 1 and 2, were permanently successful, in one of them the artery being left pervious, in the other, being obliterated by coagulum; that a third, Experiment 3, failed at the time of the operation for want of sufficient coagulum in the vessel, to defend it after the compresses were loosened; and further, that Dr. Jones's method saved this animal after simple pressure had not succeeded; and that one only, viz. Experiment 4, was altogether unsuccessful, the animal having in this case only died of the operation.

The conclusions which I think may be legitimately drawn from these experiments, and from what I have seen of M. Halmagrand's, (and which are strengthened by the failure of the last two, since the reasons why they failed are plain,) are the following:

1st. Experiments 1, 2, and 4, demonstrate that pressure continued for ten minutes upon an injured artery, *in a nearly open wound* of the soft parts, by means of small compresses moistened in various liquids, may check the hæmorrhage from an artery in which a longitudinal or transverse wound has been made, or from which a portion has been removed. They thus prove an additional point to the facts ascertained by Dr. Jones, whose experiments upon partially divided arteries were made in *wounds closed by a continued suture.*

2dly. Experiments 3 and 5, shew that when a portion of an artery has been *altogether removed*, the hæmorrhage may be restrained, and the vessel permanently closed, by allowing the blood to coagulate in the soft parts, as in a diffused aneurism; Dr. Jones had previously proved this point with regard to several other kinds of wounds of arteries, but had not attempted this peculiar modification of injuries of vessels.

3rdly. Experiments 1, 2, and 4, prove that the means by which hæmorrhage is controlled, when pressure is made for a short time in an *open wound*, are the same which Dr. Jones's experiments had shewn to be exerted in a *closed wound:* viz. the formation of coagula, either in the lips of the wound of the vessel, or in the cavity of the artery, or in the cellular membrane around it, or in any two of these situations, or in all of them at once; except in some longitudinal wounds, which may occasionally be closed by the deposition of lymph alone, in which case the vessel may still remain pervious.

4thly. It is shewn further, that if coagulation takes place imperfectly, or if the clot is suddenly or violently disturbed, immediate hæmorrhage will occur.

In two of M. Halmagrand's experiments the compresses were probably thrown off thus by the exertions of the sheep, and the coagulum being consequently disturbed, both of them died. In Experiment 3, also, I could not obtain coagulation at all, the animal being so very restless on the table, and probably it would have died, had I not produced coagulation in a different way.

5thly. Experiments 4 and 5, shew that although the hæmorrhage may be controlled for a time, it may subsequently return at various periods from the causes pointed out in the last section as capable of producing immediate hæmorrhage. In Experiment 4, bleeding commenced about twenty or twenty-four hours after the injury, in Experiment 5 on the third day, and in one of M. Halmagrand's experiments at the London Hospital, described in the Medical Gazette, it did not occur till the fifth day after the wound. Dr. Jones's experiments also lead us to the same conclusion with regard to secondary hæmorrhage from wounded arteries.

6thly. It is probable that styptics applied to a bleeding artery can only be beneficial in two ways,—first, by causing contraction of the coats of the bleeding artery. It is proved, however, by these experiments, as well as by Dr. Jones's and M. Halmagrand's, that whatever may be their effect upon *small* arteries, styptics scarcely induce any contraction of the coats of a *large* artery, and often none at all: but, that whatever may be the means employed to control the hæmorrhage, the formation of a clot, and the deposition of lymph, are almost always necessary. Surgical experience, moreover, teaches us the same lesson with regard to partially divided arteries, although, no doubt, in cases where an artery is wholly divided, contraction is powerfully exerted to prevent loss of blood. Or, SECONDLY, styptics may be of service by accelerating or promoting the coagulation of the blood. If, for instance, the decoction of galls, which was employed in the first experiment, be mixed with recent blood, it instantly produces coagulation, and such, I am informed, is also the effect of MM. Talrich and Halmagrand's 'Liquide Hæmostatique;' but Ruspini's styptic, which was permanently successful in the second experiment, and which is often employed in practice with marked benefit, produces no coagulation, nor any other sensible effect on recent blood; neither, of course, does plain water, which was temporarily successful in Experiment 4, promote coagulation. If, then, pressure for a few minutes upon a wounded artery can permanently prevent hæmorrhage, when that pressure is made with compresses dipped in various fluids, which neither produce contraction of the artery, nor facilitate coagulation, we are justified in concluding that if styptics *are* employed, the cessation of the hæmorrhage is to be ascribed principally, if not entirely, to the pressure, or at all events in a minor degree only to the action of the styptic.

7thly. These experiments further demonstrate that the effects of pressure with or without the use of styptics are precarious and uncertain, even in brutes, and therefore, *à fortiori*, ought not to be depended on in cases of wounded arteries in man, to the exclusion of the almost uniform success of the ligature, whenever its employment is practicable. And, in fact, every day's experience teaches us, in the most striking manner, the important lesson, which these and all other experiments on the subject corroborate, that wherever there is an external wound communicating directly with a large artery, although 1st, there may be complete cicatrization of the wound, and consequently no further hæmorrhage; or 2dly, that a circumscribed false aneurism *may* be formed, (this may take place at least in man, though I am not aware that an aneurism has ever been formed in any experiments upon brutes,) which is capable of being cured by the common operation of tying the artery at some future time, at a distance from the wound; yet we cannot feel confident that either of these circumstances will ensue, but, on the contrary, are obliged to entertain constant apprehension lest the barrier afforded by the internal or external coagula should give way, and, in the first place, when the wound in the skin HAS united, a diffused aneurism should be formed requiring a ligature above and below the aperture in the vessel, but accompanied with an extensive suppurating wound, which might have been avoided if the vessel had been tied in the first instance; or, in the second place, if the skin HAS NOT united, then we cannot but feel constant anxiety lest our patient be destroyed by a sudden gush of blood, or, as more frequently happens, be worn out by irritation and by loss of blood, from the no less fatal influence of frequently repeated smaller hæmorrhage.

8thly. But lastly, while I deprecate most strongly any reliance on the use of styptics in wounds of *large* arteries, I do not deny that they may sometimes be of service as *auxiliaries* in the case of hæmorrhage from *smaller* vessels; and if further experience shall prove that the 'Liquide Hæmostatique' of MM. Talrich and Halmagrand is really superior in this respect to other styptics, I shall have as much pleasure in acknowledging the fact, as I have in ascribing to their distinguished countryman, Paré, the revival of the still greater security against hæmorrhage afforded by the ligature." 135.

III. ON THE PHENOMENA AND APPEARANCES FROM PARTIAL OBSTRUCTION IN THE CEREBRAL CIRCULATION. By *John Howship*, Esq.

Mr. Howship introduces two cases to the Society's notice with the following remarks.

" The notes from which this present paper is taken regard two cases that fell under my notice. In the first are seen the symptoms during life, and appearances after death, peculiar to inflammation of the large veins upon the pia mater; in the second may be observed the inconvenience occasioned by the pressure of a large tumour, so situated as to prevent the free return of venous blood from the affected side of the head; and in both are shewn the consequences induced by a partial obstruction to the flow of blood through the brain. In the one I shall demonstrate a particular appearance in the medullary substance of the brain, which although more than once previously met with, I had not before been able satisfactorily to explain; and in the other, we shall have occasion to remark (and that to a very curious extent) the extraordinary powers a large artery may sometimes acquire and exert in a very infirm constitution, when labouring to drive forward the blood through the round of an impeded circulation." 424.

*Case* 1. J. E., a dissolute young woman, miscarried on May 17th, 1830. On the 21st she was brought into the St. George's Infirmary. On the following day and night she was very languid—had no pain—had some thirst with quick pulse—slight stupor. This, with difficulty of articulation, gradually increased, so that for several days before she died she was quite unable to speak. During her illness she frequently moaned aloud—wanted brandy and gin, to which she had been accustomed—had slight tremors and occasional subsultus. For some days before her death the right side was paralytic, and she passed her stools involuntarily. She died on the 29th, and was examined by Mr. Howship on the 30th.

*Dissection.* On reflecting the dura mater, an appearance of thickening and opacity was seen on the right hemisphere, from inflammation of one of the large veins. Every part of the pia mater from which blood was collected by the branches of this vein was red from inflammation, and had specks of blood effused into its fine cellular texture. The cortical and medullary substance of that hemisphere were healthy, yet exhibited the peculiar appearance of every capillary within a given space being enlarged to three or four times its natural diameter. On the left hemisphere two or three of the large veins had become inflamed, and there were small effusions of blood in minute masses between the pia mater and brain. On cutting into the cerebral substance the congested capillaries had apparently ruptured, depositing within the medullary substance small coagula, a few grains each in weight. There was nothing unnatural in the thorax or abdomen.

" OBSERVATIONS.—The above appearances may be regarded, I conceive, as demonstrative of the effects induced by congestion, affecting a part of the brain, from inflammation and obstruction in the trunk of the corresponding vein. The more extensively diffused effects of general congestion upon the surface, or within the substance of the brain, proceeding from various causes, have been frequently enough observed; but the more partial consequences incident to obstruction, of one or two only of the superficial veins, have been less correctly noticed.

I have presented two portions of the left hemisphere of the cerebrum, which exhibit these appearances very distinctly." 427.

*Case* 2. In December 1818, Mrs. J. then aged 77, consulted Mr. Howship for a tumor in the neck, which appeared to be an enlargement of the right lobe of the thyroid gland. The swelling had commenced at the age of 25. Mr. Howship tried the burnt sponge, but it so disturbed the pulse and nervous system that it was of necessity abandoned. In June 1825, Mr. H. was again consulted. The lady was then subject to paroxysms of convulsive cough, orthopnœa, and great languor. The tumor was now of the size of a small melon. The right carotid artery pushed outwards by the tumor, pulsated, as did all the arteries on that side of the neck, with considerable activity. In 1828, there was superadded difficulty of swallowing, and more dyspnœa. In 1830, Mr. Howship found the right carotid beating with extraordinary force and activity, the coats apparently thickened, and the diameter of the vessel increased. The trachea and larynx were pressed to the left side. On the 18th April, 1831, she was attacked with giddiness and loss of memory; in the night she lost sensation and motion in the left side and extremities. By leeching and cathartics she was relieved, but early in the morning of the 19th she died.

"*Dissection.* In the head, between the arachnoid membrane and pia mater, there was a considerable serous effusion, most conspicuous over the right hemisphere. On gently separating the hemispheres, the corpus callosum was apparently tense; and about ℥iss. of serum were found in the ventricles; the septum lucidum being expanded and thin. The substance of the corpus callosum towards its right margin, was somewhat softer than natural; the medullary matter cut into, being found to contain several cavities or small spaces within its substance, filled with serous fluid.

I removed the tumour from the neck; and on subsequent examination found the internal jugular vein, in company with the artery, passing round the tumour; it was now also ascertained by measurement, that although the right carotid artery was very much larger and thicker, it was not materially longer than the left; a circumstance explained by the unusual length of the arteria innominata, in this instance.

It was pretty evident that the tumour itself contained some fluid; but desirous to preserve its figure entire, I preferred immersing it in rectified spirit, without making any division of its substance, in which state it is, therefore, now put up." 432.

There were some other unimportant appearances in the abdomen. Let us hear what Mr. Howship has to say upon the case.

"Observations.—The protracted continuance of the disease in the above case presents only that character with which we are sufficiently familiar. It is not, however, common to see the trunk of the carotid artery so displaced, as to be found directly beneath the integuments of the neck, and apparently extended so as to occupy nearly half the circumference of a large tumour. But the most unusual circumstances, perhaps, are the very peculiar increase in the size and strength of that vessel, and most especially the extreme force with which it laboured to overcome the difficulties with which it had to struggle. In these last particulars, this case affords a very rare example in illustration of a principle laid down by the late Mr. Hunter, which disposes all muscular parts to acquire increased strength and power, equivalent to any incidental difficulty or embarrassment that may arise in the performance of their functions, from the inroads of disease. 433.

We do not conceive it an uncommon thing to see arteries, large or small, that supply growing tumors, become enlarged. Perhaps it is rare to find the carotid so much affected as in this instance. Neither do we see aught very strange in the serous effusion in the brain. It is rather to be regretted that Mr. Howship did not particularize the condition of the inflamed veins in the first case. On the whole, we confess that we do not derive any great information from the cases, but that may be our own misfortune or fault.

### IV. Case of Ovarian Disease, complicated with Pregnancy. By *Thomas Hewlett*, Esq.

This is a long but extremely interesting case. We will abbreviate it as much as possible.

A lady, æt. 36, who had borne six children without difficulty, being now in her seventh month of pregnancy, sent for Mr. H. on the 25th of August last. She had pains returning at intervals. He discovered on examination a tumour occupying the hollow of the sacrum, and allowing little more than a finger to pass between it and the pubes; he could feel no os uteri, and there was no discharge. He drew blood, and ordered an aperient. The pains returned in three days, bore an uterine character, and were only controlled by opiates. Mr. H. was undecided, and, the constitution sympathising, Dr. Locock was called in on the 21st September.

"After a very patient and careful examination per vaginam, he thought he could reach a *segment* of the os uteri high up above the pubis. With regard to the swelling, we both agreed that it bore more resemblance to an ovarian tumour than what I at first thought it was, viz. a partially retroverted uterus. On passing the hand over the abdomen, the boundary of the womb could be distinctly traced; and in the left hypochondriac region, distinct from uterus, a hard unyielding substance, apparently floating in fluid, could be plainly felt. This Dr. Locock at once pronounced ovarian." 228.

The legs were œdematous. Saline purgatives, quiet, opiates, and puncturing of the legs were resorted to with relief. On the 6th October the pains returned with difficulty of voiding urine. No os uteri could be found. On the 7th, Drs. Locock and Merriman arrived. The latter reached with difficulty what he believed to be a *part* of the os uteri, and the extremity of the finger brought away with it a small quantity of dirty-looking secretion. He was inclined to think the case one of partial retroversion, and recommended opiates, &c. with the hope of getting the lady to her full time. The sickness was now distressing, the breathing oppressed, the lower limbs distended. The motions of the fœtus ceased on the 8th. On this, the 9th, and 10th, she had a slight discharge, such as had appeared on Dr. M.'s finger; on the 12th the discharge was purulent. Examination shewed little difference. On the evening of the 11th the pains were severe; next morning they ceased, and a large discharge of liquor amnii followed, without perceptible alteration of the parts. At 10, p. m., uterine pains returned—in the morning of the 13th she had the regular grinding pains of labour—they increased till the evening—at 7, p. m., there was not the smallest alteration of the parts, no os uteri perceptible, no mobility of the tumor. At half-past seven there was a different kind of pain—on attempting to introduce the catheter, there was a new and unusual difficulty. When the water was drawn off Dr. Locock again examined. He announced that the head was fast descending. The improvement had taken place between 7, p. m. and 8, p. m. At 10, p. m, the fœtus was expelled, and immediately afterwards the placenta; the child was putrid; the bones of the head broken up. The tumor was now discovered on the right side, as before delivery, and the left ovarian tumor, which could be distinctly felt, had descended in the abdomen.

In the evening after delivery the abdomen was found as large as ever, partly from an accumulation of fluid, chiefly from a large tumor occupying the whole left side of the abdomen. Purgatives and salines were given, and little alteration occurred till the 17th. There were then what the patient called after-pains, some dyspnœa, some increase of size in the abdomen. At 2, a. m. of the 18th, more dyspnœa, pain in the hypogastrium, sickness, fever. She was bled and had an opium suppository. The blood was buffed and cupped, and though a temporary alteration was obtained, it was thought necessary to repeat the bleeding, with again some relief. Circumference of the abdomen 38 inches.

She passed a very bad night, the symptoms continuing. On the 19th the abdomen measured 41 inches, and, to give relief, Mr. H. drew off with the trocar the abdominal fluid, amounting to five pints and a half of serum. She was relieved, but only for a time. The sickness and other symptoms returned; hydrocyanic acid mitigated the former. On the 22d the symptoms were lulled, but the calm was that preceding dissolution. She became insensible, and in the morning of the 23d she died.

*Dissection.* "The body was examined seven hours after death, before decomposition could have effected any change in the appearance of diseased parts. The upper part of the body was much emaciated. The lower extremities œdematous. On turning back the abdominal parietes, a large solid diseased mass, having a *fungoid appearance*, and lying on the left side, presented itself. On tracing this, it was found to be the left ovary, lying in the left iliac fossa and ascending to the diaphragm, having that form of disease termed by Dr. Seymour 'malignant tumour of rapid growth.' On the right side, and lying in the pelvis, was the other ovary affected with the same disease, and forming the tumour which was felt before, and so much obstructed, the birth of the child: it entirely filled the hollow, and was very firmly attached to the bones of the pelvis. To the front, and resting on the tumour, was the uterus, being free from disease; behind which, and between the ovaries, was a portion of intestine very dark in appearance, being, as it were, strangulated by the pressure of the morbid growth. The intestines in the neighbourhood of the tumour, bore marks of inflammation, in some parts severe. The pyloric end of the stomach was thickened, and in a state of scirrhus; the pancreatic glands much enlarged and indurated; the liver healthy in appearance; the gall-bladder distended, and having in it a single calculus: the kidneys very much enlarged, the substance of each containing ill-formed pus of a strumous character. The ureters enormously dilated, each admitting, with facility, the tip of the little finger; this appearance being readily accounted for by the pressure of the tumours, and consequent detention of the urine in its passage to the bladder; the bladder healthy, and containing about two ounces of urine. There were about two pints of serum in the cavity of the abdomen; the left side of the chest contained about four ounces of serum; the heart, large vessels, and lungs being healthy. The head was not examined.

It is an interesting and important fact, in relation to the growth of the tumours, that I had attended this lady in two previous confinements, the last of which took place just eighteen months previous to the present one, and that on neither occasion was there any symptom to warrant the supposition that the ovaries were diseased, her 'getting up' from each being uninterruptedly good. Her health also, during the first seven months of her recent pregnancy, was unusually good; although, from watching at the sick bed of her mother, and other circumstances, she had had much to try it. It was observed, however, by her friends and herself, that she was larger for the period than she had ever been before.

The weight of the preparation was estimated at about seventeen or eighteen pounds." 238.

This is certainly an interesting and instructive case. It occupies 13 pages of the Society's volume; our reader's will find all the points in two. The obstetrical deductions appear to us to be simple, and such cases are only valuable as facts to be remembered in future difficulties. Such facts, when published, become the experience of the many—when confined to the man who sees them, they form the experience, and they contribute to the professional sagacity, of the individual. It is well to treasure up the recollection of difficulties past, in order that their solution may be applied to the analysis of difficulties to come.

There is one pathological circumstance not unworthy of attention. The urine was prevented from getting with facility into the bladder, or with freedom out of it, by the pressure of the pelvic tumour. The ureters became dilated, the kidneys enlarged, the urine retained in them. This is what occurs in cases of old stricture of the urethra, and, accordingly, we find the same state of kidney—"pus of a strumous character in its substance." In cases of old stricture, nothing is more common than to find the kidneys occupied with many small abscesses, containing pus of the character mentioned. It is also common to call these strumous abscesses of the kidney. The pus resembles in appearance that found in the pulmonary tubercles, in the stage of softening, and there, so far as we can see, the analogy between such an affection of the kidney and scrofula ceases. The disease appears to be clearly the result of mechanical obstruction to the flow of urine. The kidneys are loaded, impeded in their function; that is demonstrable. They then become irritated, fall into a state of chronic inflammation, become unnaturally vascular, and subsequently studded with partial formations of pus. Chronic inflammation is probably the cause of these, but whether it be or be not, chronic inflammation accompanies them. We refer our readers to the valuable remarks of Mr. Brodie on calculous diseases.

The shorter remaining papers in the volume before us will be noticed in the Periscope of our present number; the longer must be deferred till our next.

